# Antibodies to MHC Class II Molecules Induce Autoimmunity: Critical Role for Macrophages in the Immunopathogenesis of Obliterative Airway Disease

**DOI:** 10.1371/journal.pone.0042370

**Published:** 2012-08-10

**Authors:** Masashi Takenaka, Venkataswarup Tiriveedhi, Vijay Subramanian, Kiyotaka Hoshinaga, Alexander G. Patterson, Thalachallour Mohanakumar

**Affiliations:** 1 Department of Surgery, Washington University School of Medicine, St. Louis, Missouri, United States of America; 2 Department of Pathology and Immunology, Washington University School of Medicine, St. Louis, Missouri, United States of America; 3 Department of Urology, Fujita Health University School of Medicine, Toyoake, Aichi, Japan; Children's Hospital Boston, United States of America

## Abstract

Previous studies have shown that intrabronchial administration of antibodies (Abs) to MHC class I resulted in development of obliterative airway disease (OAD), a correlate of chronic human lung allograft rejection. Since development of Abs specific to mismatched donor HLA class II have also been associated with chronic human lung allograft rejection, we analyzed the role of Abs to MHC class II in inducing OAD. Administration of MHC class II Abs (M5/114) to C57BL/6 mice induced the classical features of OAD even though MHC class II expression is absent *de novo* on murine lung epithelial and endothelial cells. The induction of OAD was accompanied by enhanced cellular and humoral immune responses to self-antigens (Collagen V and K- α1Tubulin). Further, lung-infiltrating macrophages demonstrated a switch in their phenotype predominance from MΦ1 (F4/80^+^CD11c^+^) to MΦ2 (F4/80^+^CD206^+^) following administration of Abs and prior to development of OAD. Passive administration of macrophages harvested from animals with OAD but not from naïve animals induced OAD lesions. We conclude that MHC class II Abs induces a phenotype switch of lung infiltrating macrophages from MΦ1 (F4/80^+^CD11c^+^) to MΦ2 (F4/80^+^CD206^+^) resulting in the breakdown of self-tolerance along with an increase in autoimmune Th17 response leading to OAD.

## Introduction

Lung transplantation is currently employed as a treatment option for patients with end-stage pulmonary dysfunction. Chronic rejection manifested as bronchiolitis obliterans syndrome (BOS) represents the leading cause of long-term allograft failure in transplant recipients [1], [2]. Multiple immune and nonimmune mechanisms have been proposed to contribute to the pathogenesis of chronic rejection resulting in a slow and progressive deterioration of allograft function over months to years [3], [4]. Histopathologically, chronic rejection is an inflammatory process resulting in replacement of allograft parenchyma with fibroproliferative changes eventually resulting in occlusion of small airways in the allograft [5].

Several studies have suggested that allorecognition of mismatched donor histocompatibility antigens (HLA) is critical for the pathogenesis of chronic allograft rejection [6], [7]. Clinical and experimental evidences have documented the role of both T and B-cell-dependent immune mechanisms for the pathogenesis of chronic rejection [8], [9]. Antibodies (Abs) directed against mismatched donor histocompatibility antigens have been shown to develop during the post-transplant period following kidney, heart, and lung transplantation and has been shown to correlate with both acute and chronic rejection [10], [11], [12]. Allo-Abs can induce graft injury either directly or indirectly [13]. Specific binding of the Abs to MHC can result in the activation of lining cells such as endothelial or epithelial cells leading to the secretion of growth factors, chemokines, and cytokines which favor the recruitment of inflammatory cells (macrophages, NK cells and PMNs) to the graft, contributing to graft damage [14], [15], [16]. The high levels of fibrogenic growth factors in the setting of a proinflammatory microenvironment induces proliferation of fibroblasts and smooth muscle cells leading to tissue remodeling and subsequent luminal obliteration of tubular structures in the graft, a hallmark of chronic rejection [16].

Our studies in lung transplant patients who develop BOS indicated that the host immune system is primed to recognize both donor-specific HLA class I and II peptides [17], [18]. Moreover, the development of donor-specific antibodies to HLA demonstrated a significant correlation with the development of chronic rejection following human lung transplantation [19]. Studies have also shown that development of Abs to donor HLA class I precedes the development of BOS in human lung transplant recipients [19], [20]. In addition to Abs to HLA class I there are reports demonstrating a significant correlation between the development of Abs to mismatched donor HLA class II antigens and development of BOS [21]. These results strongly support the concept that *de novo* development of Abs to donor HLA following transplantation can contribute significantly to the pathogenesis of BOS following human lung transplantation. Based on this we proposed that Abs to HLA as well as other risk factors including cellular rejection, primary graft dysfunction, viral infections and gastroesophageal reflux, etc can activate inflammatory cascades which will expose the antigenic epitopes of self-antigens (self-Ags) leading to the development of an immune response to self-Ags leading to chronic rejection following lung transplantation [4], [22], [23], [24].

Therefore, with a goal to specifically address the role of alloimmune responses in the development of OAD, we developed a murine model for OAD, wherein MHC class I Abs were intrabronchially administered into mice [25]. In this model, the animals developed OAD lesions as manifested by epithelial hyperplasia, cellular infiltration, luminal occlusion and fibrosis around the smaller bronchioles and developed both cellular and humoral immune responses to lung associated self-Ags, K-α1 Tubulin (Kα1T) and Collagen V (ColV) [26], [27]. However, the question remained whether Abs to MHC class II can also elicit OAD lesions since murine endothelial and epithelial cells *de novo* do not express MHC class II antigens. In this communication, we report that intrabronchial administration of Abs to MHC class II molecules also induced obliterative airway disease (OAD) lesions in C57bl/6 mice in spite of the fact that murine airway epithelial and endothelial cells naturally don't express MHC class II [28]. We demonstrate that resident macrophages play a central role in the development of MHC class II mediated OAD development as evidenced by macrophage phenotype shift from MΦ1 to autoimmune MΦ2, along with breakdown of peripheral tolerance and precipitous increase of Th17 leading to OAD.

## Results

### Intrabronchial administration of MHC class II Abs induced the development of OAD lesions

**Figure 1:**
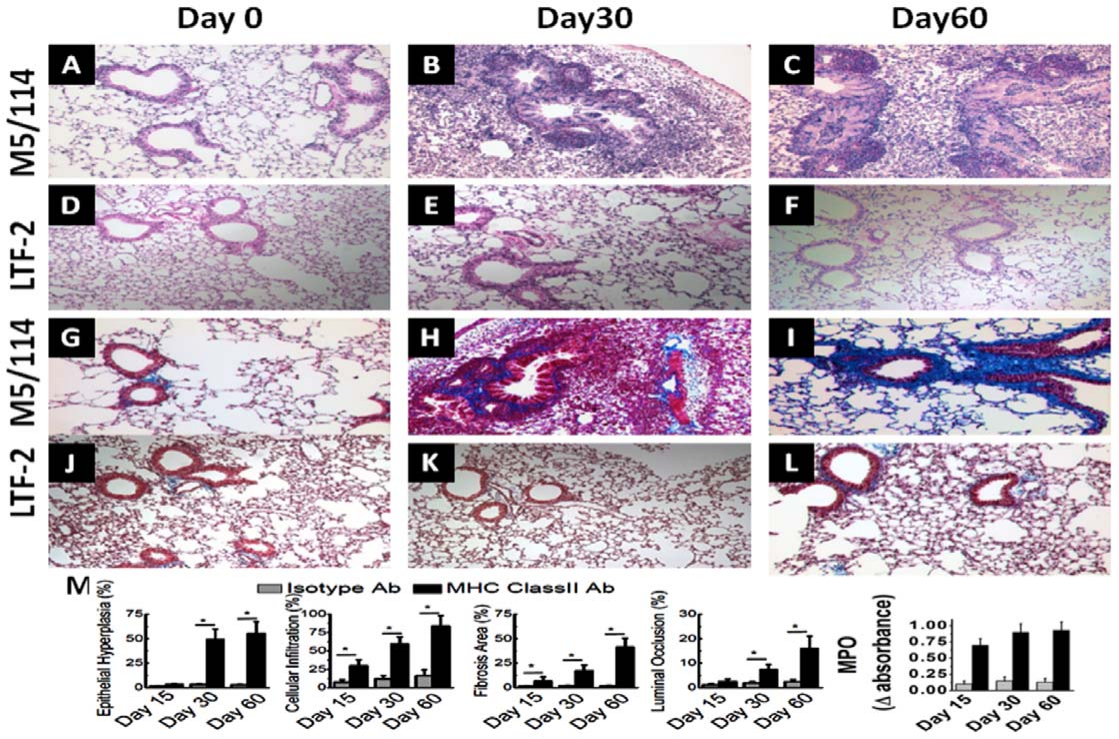
Histology from lungs harvested on day 0, 30 and 60 following intrabronchial administration of M5/114 (MHC class II Ab) or LTF-2 (isotype control) to C57BL/6 mice on day 1,2,3,6 and weekly. (A–F) represent lungs analyzed by H&E staining; (G–L) represent lungs analyzed by trichrome staining; and (M) represents morphometric analysis performed using NIS-Elements BR software to quantitate the epithelial hyperplasia, cellular infiltration, fibrosis, luminal occlusion and neutrophil infiltration (assayed by myeloperoxidase activity). The data is represented as a mean ± SEM over a 5 different animals in each group, the significance (*p*-value <0.05) was determined by student-t-test and represented with an asterisk (*).

**Figure 2:**
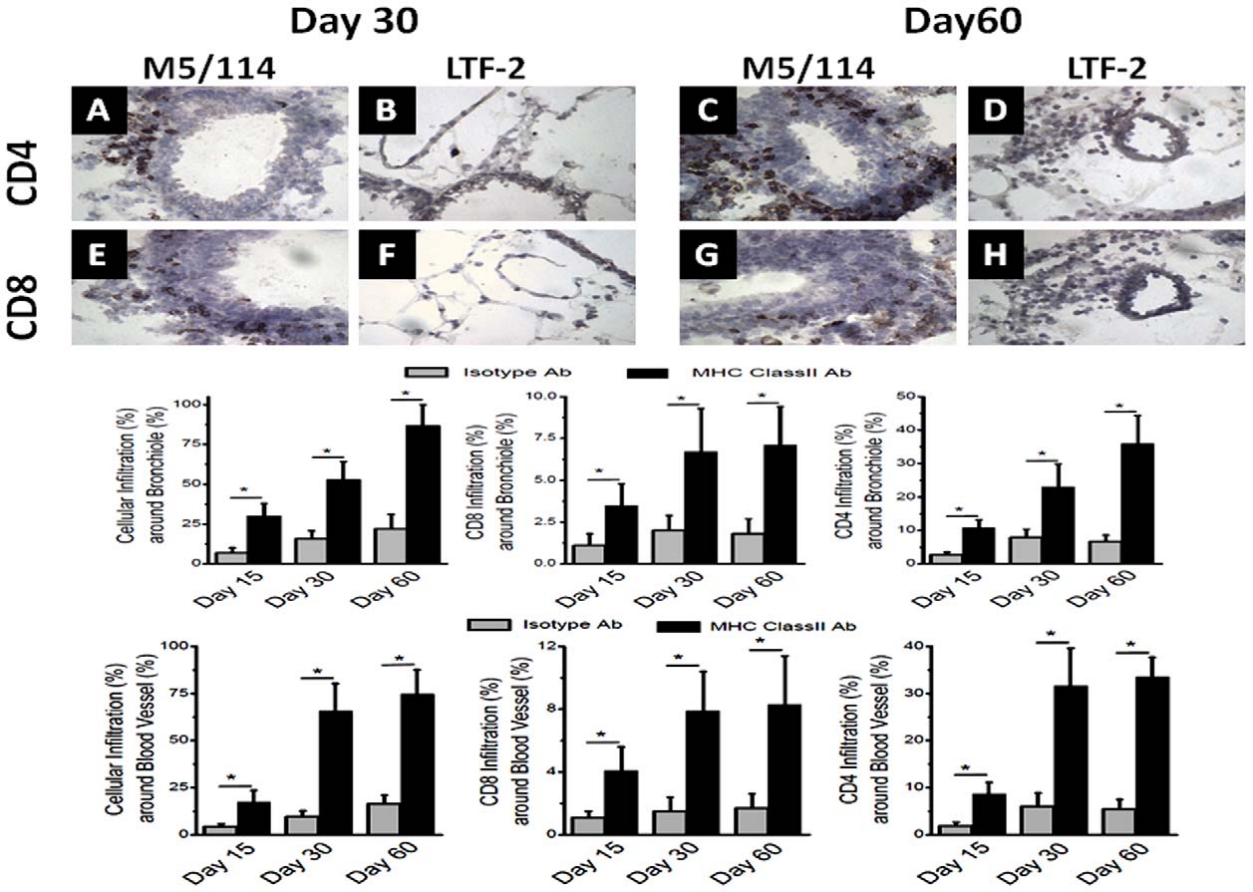
Immunohistochemistry from lungs harvested on day 30 and 60 following intrabronchial administration of M5/114 (MHC class II Ab) or LTF-2 (isotype control) to C57BL/6 mice. (A–D) represent staining for CD4+ T cells; (E–H) represent staining for CD8+ T cells; (I and J) represent morphometric analysis to quantitate the total cellular infiltration, CD8 infiltration, CD4 infiltration on day 15, 30 and 60 around bronchioles (I) and blood vessels (J). The data is represented as a mean ± SEM over a 5 different animals in each group, the significance (*p*-value <0.05) was determined by student-t-test and represented with an asterisk (*).

We employed a murine model of OAD, wherein intrabronchial administration of anti-MHC class I Ab (anti-H2K^b^) resulted in the development of classic OAD lesions in the lungs of C57BL/6 mice as evidenced cellular infiltration around the vessels, bronchioles, epithelial hyperplasia and fibrosis [25]. In the current study, we determined whether administration of MHC class II Abs can also result in OAD lesions in native lungs since endothelial and epithelial cells naturally don't express MHC class II on the cell surfaces. We administered M5/114, a monoclonal Ab (mAb) specific to I-A and I-E expressed on C57BL/6 animals or isotype control Abs (IgG2b) intrabronchially into male C57BL/6 mice (6–8 weeks old) on days 0,1,2,3, 5 and weekly thereafter. The animals were sacrificed on day 30 and 60, and lungs were harvested for histological analysis. As shown in figure 1, morphometric analysis of sections stained by H&E (Fig. 1A–1F) and trichrome (Fig. 1G–1L) of the lungs on day 0, 30 and 60 following administration of anti-MHC class II demonstrated significant luminal occlusion (day 30: Isotype = 1.9±0.6%; class II = 7.3±2.1%; day 60: Isotype = 2.4±0.9%; class II = 15.9±5.2%; p-value<0.05) and epithelial hyperplasia (day 30: Isotype = 3.1±0.5%; class II = 49.1±10.7%; day 60: Isotype = 2.9±0.6%; class II = 55.2±12.3%; p-value<0.05). Similarly, morphometry of trichrome staining demonstrated significant fibrosis (day 30: Isotype = 1.7±0.6%; class II = 17.1±5.9%; day 60: Isotype = 1.6±0.7%; class II = 41.4±9.1%; p-value<0.05) in MHC class II Ab administered animals. Cellular infiltration results shown in Figure 1M also demonstrated increased cellular infiltration (day 30: Isotype = 11.9±4.3%; class II = 59.4±9.7%; day 60: Isotype = 16.1±8.1%; class II = 83.7±14.3%; p-value<0.05). Predominant cellular infiltration was seen around the bronchioles (day 30: Isotype = 15.7±5.1%; class II = 52.7±11.3%; day 60: Isotype = 21.9±9.2%; class II = 86.5±13.4%; p-value<0.05) and blood vessels (day 30: Isotype = 9.6±3.1%; class II = 65.9±14.4%; day 60: Isotype = 16.3±4.7%; class II = 74.7±12.9%; p-value<0.05) (Figure 2). Results presented in figure 2A–D demonstrated that the CD4 T cell infiltration was markedly increased around the bronchiole (day 30: Isotype = 7.9±2.5%; class II = 23.1±6.7%; day 60: Isotype = 6.7±1.9%; class II = 35.9±8.4%; p-value<0.05) and blood vessels (day 30: Isotype = 6.0±2.9%; class II = 31.7±7.9%; day 60: Isotype = 5.4±2.1%; class II = 33.6±4.1%; p-value<0.05) in MHC class II Ab treated animals. Similarly, an increase in the CD8 T cell infiltration (Figure 2E–H) around the bronchiole (day 30: Isotype = 2.0±0.9%; class II = 6.7±2.6%; day 60: Isotype = 1.8±0.9%; class II = 7.1±2.3%; p-value<0.05) and blood vessels (day 30: Isotype = 1.5±0.9%; class II = 7.9±2.5%; day 60: Isotype = 1.7±0.9%; class II = 8.3±3.1%; p-value<0.05) was also noted in MHC class II Ab treated animals. These results demonstrates that intrabronchial administration of MHC class II resulted in OAD lesions in the murine model even though the epithelial and endothelial cells naturally don't express MHC class II molecules on the cell surface.

### Administration of MHC class II Ab enhances the development of humoral and cellular responses to lung associated self-Ags (Kα1T and ColV)

**Figure 3:**
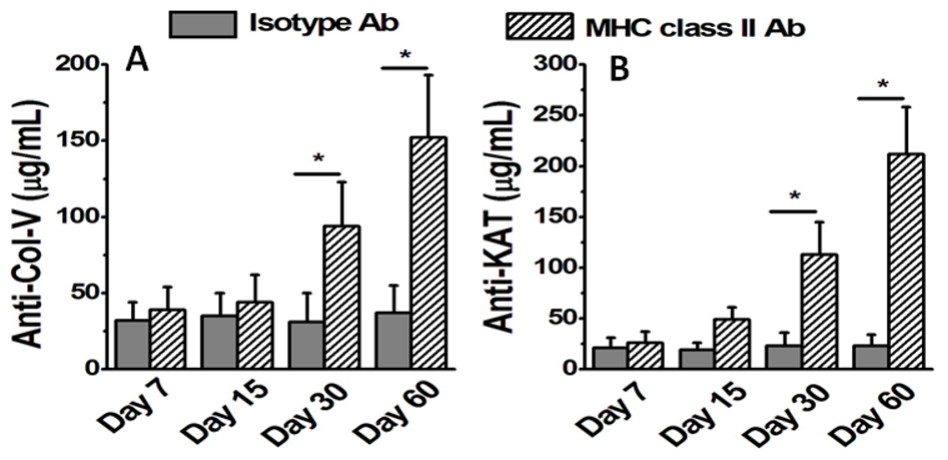
Analysis of the humoral immune response on serum collected from day 7, 15, 30 and 60 following administration of MHC class II Abs or isotype C57BL/6 animals. (A,B) represent the serum concentration of Abs to self-Ags ColV (A) and Kα1T (B) analyzed by ELISA. The data is represented as a mean ± SEM over a 5 different animals in each group, the significance (*p*-value <0.05) was determined by student-t-test and represented with an asterisk (*).

Abs to MHC class I molecule has been shown to induce the development of immune responses to lung associated self-Ags mainly ColV and Kα1T both in the murine model of OAD and in BOS following human lung transplantation [19], [25] To determine if Abs to MHC class II can also induce immune responses to lung associated self-Ags sera from mice following administration of anti-MHC class II were tested by ELISA and concentrations of Abs to ColV and Kα1T were measured on days 7,15,30 and 60. As shown in figure 3, development of Abs to ColV (day 30: Isotype = 31±19 µg/mL; class II = 94±29 µg/mL; day 60: Isotype = 37±18 µg/mL; class II = 152±41 µg/mL; p-value<0.05) and Kα1T (day 30: Isotype = 23±13 µg/mL; class II = 113±32 µg/mL; day 60: Isotype = 25±11 µg/mL; class II = 212±46 µg/mL; p-value<0.05) were noted following anti-MHC class II administration that was significantly increased on days 30 and 60. This demonstrates induction of immune responses to self-Ags following administration of MHC class II Ab with the response on day 60 was equivalent to the MHC class I Ab treatment of day 30 [25].

**Figure 4:**
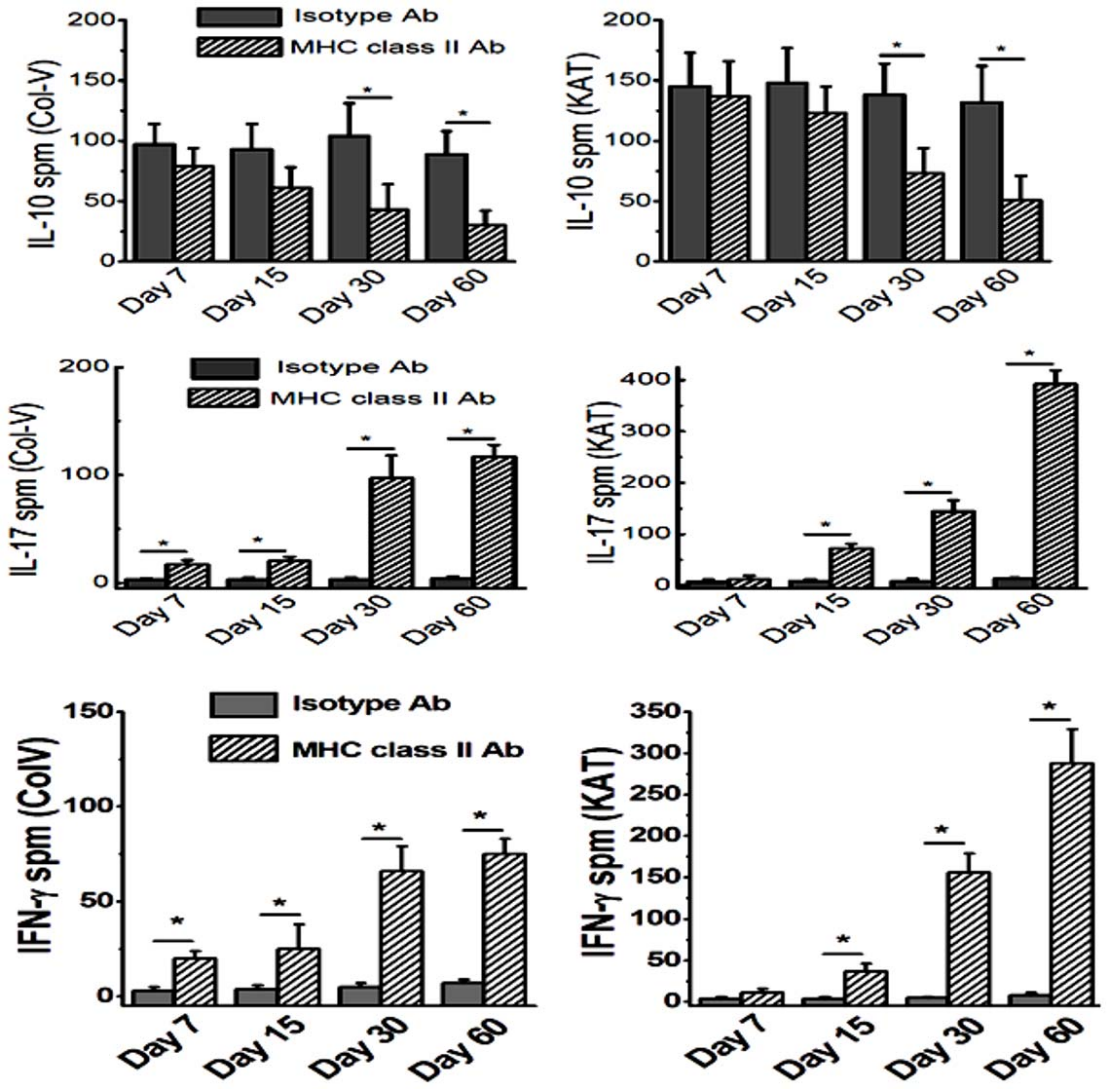
Analysis of the cellular immune response on lung infiltrating lymphocytes collected on day 7, 15, 30 and 60 following administration of MHC class II Abs or isotype C57BL/6 animals, analyzed by ELISpot. (A,B) represent the ColV (A) and Kα1T (B) specific IL-10 responses; (C,D) represent the ColV (C) and Kα1T (D) specific IL-17 responses; (E, F) represent the ColV (E) and Kα1T (F) specific IFN-γ responses. The data is represented as a mean ± SEM over a 5 different animals in each group, the significance (*p*-value <0.05) was determined by student-t-test and represented with an asterisk (*).

To determine the cellular immune responses to ColV and K-α1T we performed ELISpot on the lung infiltrating lymphocytes. As shown in figure 4A–B, anti-inflammatory IL-10 specific cellular response to ColV (day 30: Isotype = 104±27 spm; class II = 43±21 spm; day 60: Isotype = 89±19 spm; class II = 30±12 spm; p-value<0.05) and Kα1T (day 30: Isotype = 138±26 spm; class II = 73±21 spm; day 60: Isotype = 132±30 spm; class II = 51±20 spm; p-value<0.05) significantly diminished by day 60. On the other hand, pro-inflammatory IL-17 responses (Figure 4C–D) to ColV (day 30: Isotype = 3±2 spm; class II = 97±21 spm; day 60: Isotype = 4±2 spm; class II = 117±11 spm; p-value<0.05) and Kα1T (day 30: Isotype = 9±4 spm; class II = 144±22 spm; day 60: Isotype = 13±2 spm; class II = 392±27 spm; p-value<0.05) significantly increased by day 60. Similarly, IFN-γ response (Figure 4E–F) to ColV (day 30: Isotype = 5±2 spm; class II = 66±13 spm; day 60: Isotype = 7±2 spm; class II = 75±8 spm; p-value<0.05) and Kα1T (day 30: Isotype = 5±1 spm; class II = 156±15 spm; day 60: Isotype = 8±3 spm; class II = 288±40 spm; p-value<0.05) significantly increased by day 60. This demonstrates that upon intrabronchial administration of MHC class II Ab induces both Th17 (IL-17) and Th1 (IFN-γ) responses along with a decrease in the Th2/Treg (IL-10) responses.

### Induction of class II expression and activation of macrophages following intrabronchial administration of MHC class II Ab

**Figure 5:**
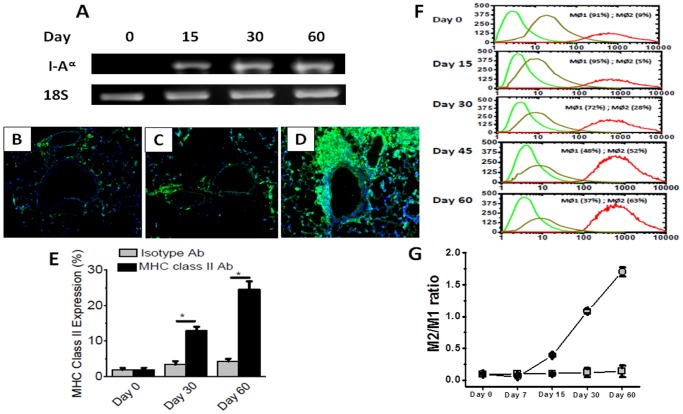
Analysis of the MHC class II expression and macrophage phenotype prior to and following the administration of MHC class II Abs. (A) represent MHC class II (I-Ak^α^) expression in the lung analyzed by PCR on day 0, 15, 30 and 60 following the administration of M5/114; (B–D) represent the immunohistochemistry on harvested lungs with MHC class II staining prior to administration of MHC class II Ab (B), day 60 of isotype administration (C) and day 60 of MHC class II Ab administration; (E) represent the morphometric quantitation of the MHC class II expression in the lung on day 0, 30 and 60 following the administration of MHC class II Ab or isotype Ab; (F, G) represent the phenotype of the lung infiltration macrophages following administration of MHC class II Ab analyzed by flow cytometry, green represent F4/80 staining (MΦ), orange represent CD11c staining (MΦ1), and red represent CD206 staining (MΦ2); and (G) represent the ratio of MΦ2 to MΦ1 macrophage phenotype on day 0, 7, 15, 30 and 60. The data is represented as a mean ± SEM over a 5 different animals in each group, the significance (*p*-value <0.05) was determined by student-t-test and represented with an asterisk (*).

MHC class II molecules are normally not expressed on the epithelial lining of native murine lungs. To check for the expression of MHC class II molecules in the lung following administration of MHC class II Ab, we performed immunofluorescence (Figure 5B–E) for MHC class II expression which demonstrated a 16 fold increase in the epithelial surface expression of MHC class II by day 60. PCR from the cDNA obtained by reverse transcription of the mRNA collected from the harvested lungs on day 0, 15, 30 and 60 also demonstrated increased expression in the MHC class II molecules (I-A^α^) on day 60 compared to day 0 (Figure 5A). We subsequently determined the role of macrophages in the development of OAD in our murine model. At first, we determined the macrophage phenotype by Flow cytometry analysis of lung infiltrating macrophages collected at day 60 following administration of either anti-MHC class II or isotype control. Results demonstrated that there is specific shift in the macrophage phenotype from MΦ1 (F4/80+ CD11c+) [29] to MΦ2 (F4/80+ CD206+) [30] at day 60. As shown in figure 5F, the M1 phenotype reduced from 91% on day 0 to 37% by day 60 following MHC class II Ab administration. Conversely, the MΦ2 phenotype increased from 9% on day 0 to 37% on day 60. The MΦ2/MΦ1 ratio (Fig. 5G) increased from 0.099±0.005 on day 0 to 1.703±0.071 by day 60 (p-value <0.05). These results strongly support that following administration of Abs specific to MHC class II molecules there is specific activation of MΦ2 macrophages which we propose to play a role in the development of immune responses to self-Ags.

### Induction of pro-inflammatory chemokines and its receptors in the lung following administration of Abs to MHC class II molecule

**Figure 6:**
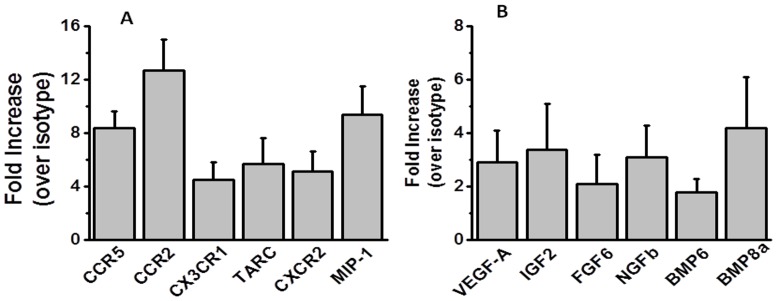
Gene expression analysis of the chemokines, their receptors and growth factors on the on the mRNA collected from the lungs harvested on day 7. The data is represented as a mean ± SEM over a 5 different animals in each group.

Gene expression analysis was done for the chemokines and its receptors. As shown in figure 6A, lungs harvested on day 7 following intrabronchial administration of MHC class II Abs demonstrated significant increase in the mRNA expression of CCR5 (8.4 fold), CCR2 (12.7 fold), CX3CR1 (4.5 fold), TARC (5.7 fold) and MIP-1 (9.4 fold) over isotype treated animals. Expression of the growth factors (figure 6B), VEGF-A (2.9 fold), IGF2 (3.4 fold), FGF6 (2.1 fold), NGFb (3.1 fold), BMP6 (1.8 fold) and BMP8a (4.2 fold) were also significantly increased over isotype animals. Other CXC and CC chemokines (CXCL13, CCL 2,7,8, MIP-3 *etc*) showed only marginal or no increase at all and did not achieve statistical significance (data not shown).

### Adoptive transfer of macrophages induced OAD lesions

**Figure 7:**
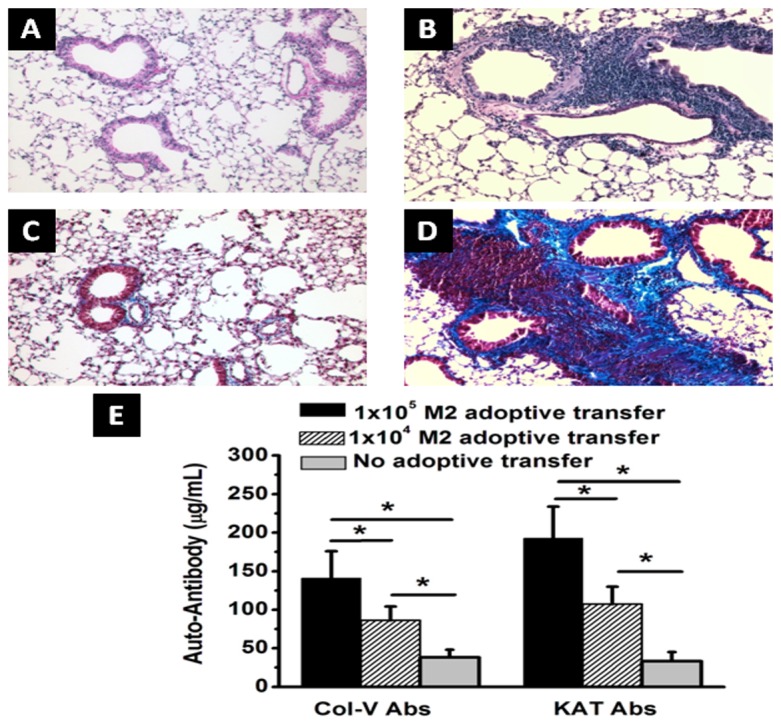
Histology from lungs harvested on day 30 following adoptive transfer of day 60 macrophages isolated at day 60 from MHC class II Ab administered mice. 0.1, and 1×10^5^ CD11b+ macrophages intrabronchially, and single intrabronchial administration of MHC class II Ab was done for adoptive transfer experiments. (A, B) H&E staining of the lungs collected from day 30 without (A) and with (B) adoptive transfer of 1×10^5^ CD11b+ macrophages following single administration of MHC class II Ab. (C, D) Trichrome staining of the lungs collected from day 30 without (C) and with (D) adoptive transfer of 1×10^5^ CD11b+ macrophages following single administration of MHC class II Ab; and (E) serum concentration of Abs to self-Ags ColV and Kα1T analyzed by ELISA. The data is represented as a mean ± SEM over a 5 different animals in each group, the significance (*p*-value <0.05) was determined by student-t-test and represented with an asterisk (*).

To demonstrate the causal role towards MΦ2 macrophages in OAD following MHC class II administration, we performed adoptive transfer of 1×10^4^ and 1×10^5^ macrophages (CD11b^+^) positively selected from lung infiltrating cells isolated from day 60 following class II Ab administration. Due to the lack of commercially available F4/80+ purification kit we performed passive transfer of CD11b+ cells from day 60 MHC class II antibody treated animals. As shown in figure 6A–D, H&E and trichrome stain analysis of the lungs harvested on day 30 following adoptive transfer of macrophages and a single dose of 200 µg/mL of M5/114 (MHC class II Ab) demonstrated development of OAD (Figure 7B and D) lesions as evidenced by luminal occlusion, epithelial hyperplasia, cellular infiltration and fibrosis by day 30 (Figure 7A and C). Similarly, adoptive transfer of day 60 macrophages (MΦ) also demonstrated increased humoral immune responses (Figure 7E) to self-Ags ColV (day 30: 1×10^5^ MΦ/single dose class II Ab = 141±35 µg/mL; 1×10^4^ MΦ/single dose class II Ab = 86±18 µg/mL; single dose class II Ab only = 38±10 µg/mL; p-value<0.05) and Kα1T (day 30: 1×10^5^ MΦ/single dose class II Ab = 193±41 µg/mL; 1×10^4^ MΦ/single dose class II Ab = 107±23 µg/mL; single dose class II Ab only = 33±12 µg/mL; p-value<0.05). These results clearly demonstrate that macrophages of the MΦ2 phenotype induces immune responses to self-Ags resulting in OAD lesions following MHC class II Ab administration in this murine model of OAD.

### Induction of Th17 differentiation of naïve CD4+ T cells by lung infiltrating macrophages

**Figure 8:**
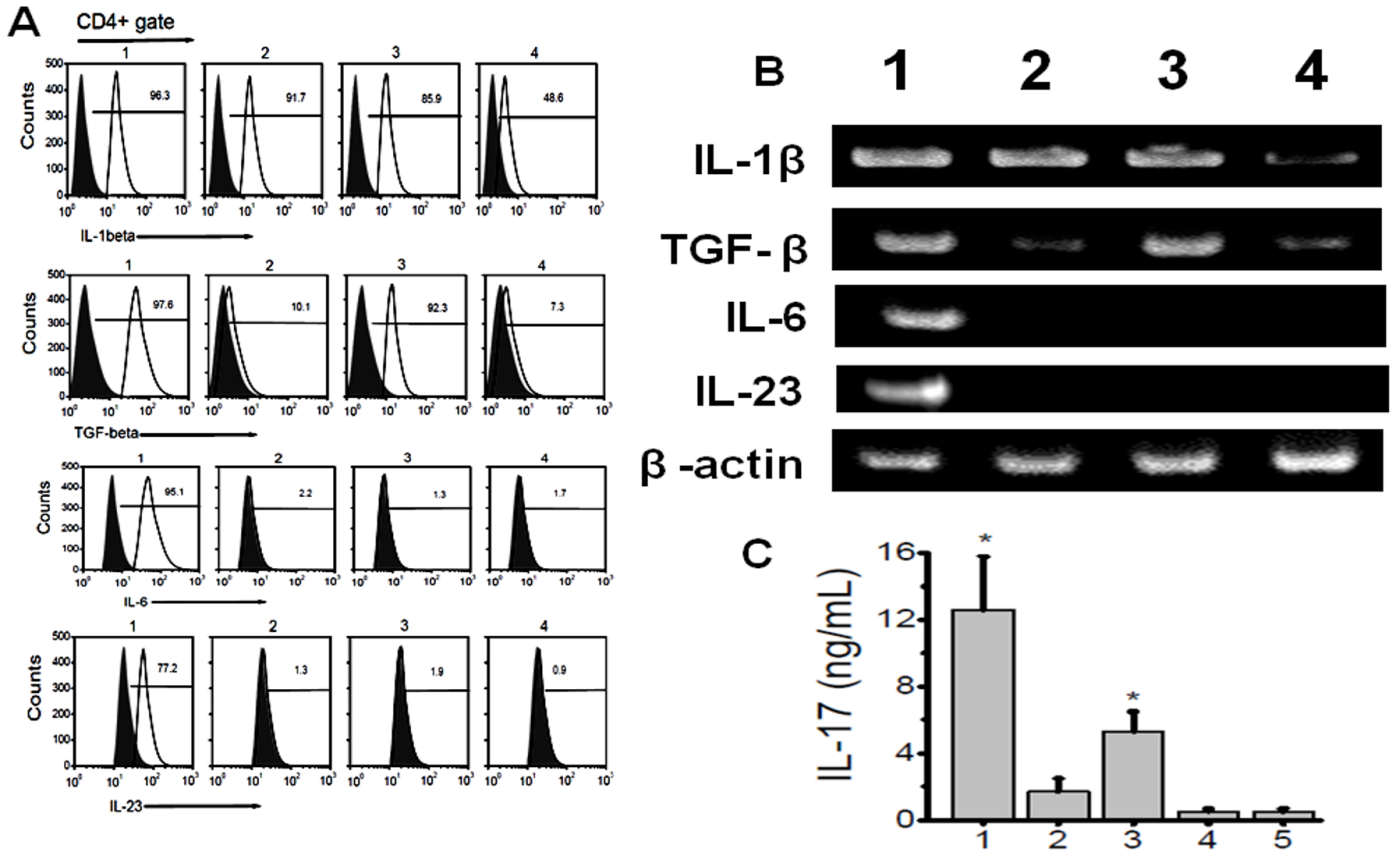
Induction of Th17 cell from naïve T cells by lung infiltrating macrophages. (A) Flow cytometry analysis for the expression of IL-1β, TGF-β, IL-6 and IL-23 cytokines on CD4+gated population; (B) gene expression of IL-1β, TGF-β, IL-6 and IL-23 in macrophages collected from lungs; (lane1) and peritoneum (lane 3) day 60 MHC class II Ab administered mice, lane 2 and 4 represent macrophages collected from lung and peritoneum of isotype administered animals. (B) represent IL-17 concentration from the media collected by co-culture of 1∶1 ratio of naïve T cells collected from splenocytes of wild type C57BL/6 mice and cultured with macrophages collected from lung (1) and peritoneum (3) of day 60 MHC class II Ab administerd mice, 2 and 4 represent co culture with macrophages collected from lung (2) and peritoneum (4) of day 60 Isotype Ab administered mice, (5 in panel B) represent lung infiltrating macrophages of day 60 MHC class II Ab administered mice in the absence of naïve T cells. The data is represented as a mean ± SEM over a 5 different experiments, the cohort with significant expression of IL-17 (p-value <0.005) compared to isotype Ab administered group (5) were represented with an asterisk (*).

The phenotypic switch in the lung infiltrating macrophages is accompanied by increased Th17 CD4+ T cells specific to ColV and Kα1T. To determine whether MΦ2 macrophages were involved in the Th17 differentiation of the naïve CD4+ T cells we performed co-culture experiments. The CD11b^+^ macrophages were isolated by MACS bead purification from the day 60 lung of MHC class II Ab administered mice. The PCR performed from the mRNA extracted from these macrophages demonstrated (Figure 8A) increased expression of IL-1β, TGF-β, IL-6 and IL-23. However, the macrophages collected from lung and peritoneum of isotype administered mice (lane2 and 4) did not show significant expression of TGF-β and IL-6. We also did not see any IL-17 gene expression by PCR in the isolated lung infiltrating macrophages (data not shown). Co-culture of lung infiltrating macrophages with naïve CD4+ T cells isolated from non-Ab administered naïve wild-type C57BL/6 mice in the presence of anti-CD3 induced (Figure 8B) significantly increased the expression of IL-17 compared to macrophages from peritoneum of MHC class II Ab administered animals as well as from macrophages from lung and peritoneum of isotype administered animals. Therefore, activated lung macrophages of MΦ2 phenotype develop following intrabronchial MHC class II Ab administration and these macrophages have the effector function to induce naïve CD4+ T cells to autoimmune Th17 phenotype.

## Discussion

The long term success of human lung transplantation is limited by the development of chronic rejection predominantly manifested as BOS that has an incidence as high as 40–70% by 5 years [5]. In spite of this high incidence, research efforts aimed at discerning the exact molecular bases of this disease have been limited predominantly due to the non-availability of an appropriate animal model [31]. This may be due to many known risk factors for chronic rejection including surgical trauma, viral infections, gastroesophageal reflux disease, primary graft dysfunction, alloimmune responses, autoimmunity etc [2]. Towards understanding this complex problem it is postulated that initial pro-inflammatory microenvironment may lead to the development of alloimmune responses to donor HLA Ags and breakdown of peripheral tolerance and development of immune responses to self-Ags (mainly ColV and Kα1T) culminating in a fibrotic cascaded leading to chronic lung allograft rejection [4], [23], [24]. Despite limitations of current animal models including heterotopic tracheal transplant model [32], murine orthotopic vascularized lung transplant model [33] and minor mismatch lung transplant model [34] there have been significant development in our understanding of the molecular and genetic basis of chronic lung allograft rejection. With a primary emphasis to study the role of alloimmunity in the development of autoimmune responses and histologic development of features of chronic lung allograft rejection, we developed a murine model [25] wherein Abs to MHC class I molecules were administered intrabronchially and upon specific binding of these Abs to lung epithelium resulted in the breakdown of peripheral tolerance to self-Ags and induced OAD (fibrosis, epithelial hyperplasia, cellular infiltration and luminal occlusion), which are considered the cardinal features of chronic lung allograft rejection. In spite of the absence of lung transplantation and non-physiologic administration of MHC class I Abs intrabronchially the ability of this model to replicate the features of chronic lung allograft rejection makes it a viable model to study the alloimmune induced autoimmunity following lung transplantation. Previous studies have demonstrated that following human lung transplantation Abs to HLA class I and II develop and are strongly correlated with the development of BOS. HLA class II unlike class I antigens have limited cell surface expression and are selectively expressed on macrophages, B-cells and dendritic cells. Native murine lung epithelium does not have expression of MHC class II molecules. However, MHC class II molecules are expressed on the alveolar macrophages [28].

In this communication, we analyzed the pathophysiological mechanisms by which Abs to MHC class II lead to the development of OAD. Following initial pilot studies with various concentrations of MHC class II antibodies (50–300 µg) we have decided to use 200 µg in 70 µL of PBS solution. We demonstrate that intrabronchial application of MHC class II Abs caused development of classic histologic features of OAD by day 60 (Figure 1 and 2). However, the development of OAD with MHC class II Abs (60 days) was delayed as compared to MHC class I Abs which occurs prior to 30 days. Similar to class I Abs, class II Abs also induced cellular immune responses to self-Ags by activation of Th17 responses and humoral immune responses to self-Ags ColV and Kα1T along with a concomitant decrease in the protective anti-inflammatory IL-10 response (Figure 3 and 4). This data is in line with previous findings in human and animal studies that towards the development of cellular mediated autoimmune responses to self-Ags there is shift in immune responses from protective IL-10 to potential lethal IFN-γ and IL-17 [22], [25], [26], [35]. This raised an interesting question as to what is the cell type to which MHC class II Abs bind to resulting in their possible activation. As shown in figure 5A, mRNA analysis demonstrated that native lung as expected had minimal expression of MHC class II molecules. However, following administration of class II Abs, by day 30 there is increased expression of MHC class II molecule in the murine lung. Furthermore, the alveolar macrophages which have intrinsic expression of MHC class II molecule undergo a phenotype switch to MΦ2 (CD206^+^) (Fig. 5B–5C), which has been attributed to be involved in the development of autoimmune responses. A convincing evidence towards the role of MΦ2 macrophages in experimental autoimmune encephalitis (EAE) has already been demonstrated wherein the macrophage/microglial cells isolated from CNS with EAE demonstrated MΦ2 phenotype [36]. Similarly, gene expression profile of peripheral blood cells from patients with new-onset systemic juvenile idiopathic arthritis revealed that the patients with this disease have increased markers of MΦ2 phenotype [37]. Additionally, around 30% of macrophages isolated from murine model for autoimmune myocarditis showed MΦ2 phenotype [38]. Our data is in agreement with the above mentioned studies wherein administration of MHC class II Abs induced activation of macrophages to autoimmune MΦ2 phenotype [38]. We also performed passive intrabronchial adoptive transfer of MΦ2 macrophages harvested from 60 days following anti-MHC class II administration induced OAD in naïve animals. Future studies with clodronate administration to specifically deplete macrophages would be needed to confirm our current data. The gene expression profile also demonstrated upregulation of macrophage specific chemokines, CCR5, CCR2, CX3CR1 [39], [40], [41]. Taken together these experiments establishes an obligatory role for macrophage activation and phenotype switch to MΦ2 phenotype in the development of MHC class II Ab induced OAD. The MHC class II knock out animals, although would specifically prove the requirement of the ligation of the MHC class II antibody to the MHC class II molecule, this however will lack the entire class II antigen presentation pathway, and consequently will not account for the other variables such as induction of IL-17/CD4+T cells, IL-6 and pro-inflammatory cytokine induced IL-17 signaling, which is one of the key factors in the development of OAD. Previous studies in our laboratory [25] using anti-keratin antibody and current studies with LTF-2 antibody (IgG2b isotype control) did not cause development of OAD lesions. These results support that specific ligation by the Fab2 fragment to the MHC molecules are needed and Fc signaling by itself may not be sufficient. However, studies with the specific Fab2 fragment of the antibodies and/or use of FcγRIIb knock out mice need to be performed to rule out the role of Fc signaling and its impact on OAD induced by anti-MHC class II.

To demonstrate for the effector role of activated MΦ phenotype in conjunction with specific antigen stimulation, we performed co-culture experiments with naïve CD4+ T-cells in the presence of lung infiltrating MΦ (Figure 8). This resulted in the differentiation of naïve CD4+T cells to IL-17 secreting T cells. In contrast, peritoneal macrophages from class II Ab administered animals and naïve macrophages from wild type both from lung and peritoneum did not induce significant Th17 differentiation. The ability of local Ag presenting cells to induce Th17 cells may therefore be an initial early stimulus in addition to the local cytokine expression and inflammatory milieu. Recent studies have demonstrated that activated lamina propria macrophages in GI tract induce Th17 CD4+T cell phenotype [42]. Similarly, IL-6 expressing dendritic cells in EAE model have been shown to induce the polarization of Th17 cells from naïve CD4 T cell precursor phenotype [43]. These data are also in agreement with our findings that activation of macrophages to MΦ2 phenotype has the ability to induce autoimmune Th17 CD4+ T cells, which we consider as the initial event leading to the development of OAD lesions.

There is also strong evidence for autoreactivity to Kα1T and ColV in the pathogenesis of chronic rejection following human lung transplant [19], [22] and in animal models of lung allograft rejection [25], [44]. Studies analyzing the immune responses to self-Ags following transplantation have clearly demonstrated that the indirect allorecognition pathway plays a crucial role [45]. In this study, analysis of the cells infiltrating the lung indicates that they are activated and secrete IFN-γ and IL-17, along with a decrease in IL-10 specific to Kα1T and ColV (Figure 4). This was followed by the development of Abs to both of these auto-Ags. It is also of interest to note that cells infiltrating the lung produced large amounts of IL-17 following stimulation with both Kα1T and ColV (Figure 3). IL-17 has been shown to be a potent proinflammatory cytokine to the local tissue environment leading to a strong proinflammatory signal that results in enhanced autoimmune response [46]. IL-17 has also been shown to play a crucial role in the induction of humoral autoimmune responses [47]. Analysis of the cellular responses to ColV in lung transplant recipients has been shown to be dependent on CD4 T cells and monocytes [35]. This response was found to be dependent on IL-6, IL-23, IL-1β, TGF-β and IL-17. The risk of BOS development was observed to correlate with the severity of the self-Ag-specific T cell responses [35]. Furthermore, in this study we demonstrate that the interaction between macrophages and IL-17 is crucial in induction of the autoimmune responses. Studies have also demonstrated that a deficiency in the IL-17 or blocking of IL-17 results in a decrease in the production of auto-Abs [48], [49]. Therefore, our finding of IL-17-producing T cells following stimulation with self-Ags indicates that these T cells provide signals toward activation of B cells, resulting in Abs to self-Ags, such as Kα1T and ColV. These results are in agreement with the report that in IL-17-deficient mice have reduced capacity to induce autoimmunity and results in lack of formation of germinal centers leading to a reduction in the titer of auto-Abs [50]. Hence, we propose that macrophage induced IL-17/Th17 polarization is a critical mediator in the induction of autoimmunity following administration of the anti-MHC class II Abs, resulting in the development of chronic rejection following lung transplant. This is in line with our previous studies on anti-MHC class I induced OAD model, where in administration of ACE inhibitors and ARBs which modulate rennin angiotensin aldosterone system (RAAS) have been shown to downregulate IL-17 signaling through tumor necrosis factor-α-dependant IL-6 and through p38/MAPKinase pathways leading to abrogation of anti-MHC-induced OAD [51]. Further, although the exact mechanisms are not clear, recent randomized control trail by Vos et al, have demonstrated that azithromycin can prevent the incidence of chronic rejection in human lung transplant recipients [52]. However the comprehensive analysis of MΦ2 signaling pathways leading to autoimmune responses and role of various drugs in abrogation of these changes leading to prevention of chronic rejection following lung transplantation needs further investigation.

In conclusion, our report demonstrates that MHC class II Abs induce OAD in a murine model for chronic lung allograft rejection. The development of OAD was strongly associated with macrophage activation to autoimmune MΦ2 phenotype. Based on this, we conclude that ligation of class II molecules expressed on alveolar macrophages by its specific Abs promote pro-inflammatory microenvironment leading to development of Th17 mediated immune responses to self-Ags leading to fibrosis and OAD resulting in chronic allograft dysfunction. Novel therapeutic strategies aimed at modifying or inhibiting the macrophage activation could aid in the prevention of development of chronic rejection following human lung transplantation.

## Materials and Methods

### Animal Studies

We utilized a murine model in which OAD, correlate of BOS, chronic rejection, was induced in the distal airways following ligation of MHC molecules in the lung by a mAb to MHC class I Ags [25]. All experiments were performed in compliance with the guidelines of the Institutional Laboratory Animal Care and Use Committee of Washington University School of Medicine (protocol 20070121). Murine mAb M5/114 specific to MHC class II Ags (both I-A and I-E, IgG2b) and isotype control LTF-2 were administered into male C57BL/6 (6–8 wk old) which has no detectable endotoxin as measured by *Limulus* amebocyte lysate assay. Abs were given at a dose of 200 µg/administration into mice. Intrabronchial administration was done as follows: each mouse was anesthetized, and the tongue was pulled out. A mosquito clamp was inserted into the mouth, and a 20-gauge catheter was placed into the trachea. The MHC class II Ab, M5/114 (200 µg) or the isotype control LTF-2 was administered into the lung on days 1, 2, 3, and 6, and then weekly thereafter.

### Histology and morphometric analysis of cellular infiltration, fibrosis and luminal occlusion

Lungs were fixed in 10% formaldehyde. Sections were cut at 5 µm thickness and stained with H&E and Masson's trichrome and analyzed under microscope Nikon ECLIPSE 55i (Melville, NY) using NIS-Elements BR software (Melville, NY). Specific CD4 and CD8 infiltration was analyzed on frozen sections treated with 3% H_2_O_2_ and EtOH for 10 min to block endogenous peroxidase activity. The sections were blocked with biotin/Avidin blocking reagent (Avidin/Biotin Blocking Kit, Vector Laboratories, Butlinghame, CA). Primary Abs were diluted using Ab dilution solution (BD Bioscienes, San Jose, CA). The sections were incubated with purified rat anti-mouse CD4 and CD8 (5 µg/mL, Santa Cruz Biotech, Santa Cruz, CA). The sections were then treated with appropriate biotin conjugated secondary Abs followed by streptavidin-HRP. The presence of positive cells was determined with diaminobenzidine substrate kit (BD Phamingen, SanDeigo, CA). Immunohistochemistry for MHC class II expression was performed using directly FITC conjugated MHC class II antibody (eBiosciences, San Diago, CA). Percentage of cellular infiltration, luminal occlusion and fibrosis was morphometrically calculated using NIS-Elements BR software (Melville, NY). Percentage of fibrosis was quantified morphometrically by addition of the total area enclosed by basement membrane at 5 different high power (40×) field and dividing by a factor of 5. Percentage of cellular infiltration and epithelial abnormalities was calculated at 5 different high power fields (40×), respectively. Slides were analyzed by random sampling after individually analyzed by two blinded professionals. The data is represented as a mean ± SEM over a 5 different measurements.

### ELISpot assay

ELISpot assays were performed on CD4+ lung infiltrating lymphocytes, as described previously [53]. Briefly, MultiScreen 96-well filtration plates (Millipore, Billerica, MA) were coated overnight at 4°C with 5.0 µg/ml capture mouse cytokine-specific mAb (BD Biosciences, San Diego, CA) in 0.05 M carbonate-bicarbonate buffer (pH 9.6). The plates were blocked with 5% BSA for 2 hrs and washed three times with PBS. Subsequently, 3×10^5^ CD4+ T cells collected from the lung by positive selction on MACS magnetic beads (Militenyi Biotec, Auburn, CA) were cultured in triplicate in the presence of ColV (20 µg/mL, BD Biosciences, San Diego, CA) or human Kα1T (10 µg/mL, from recombinant expression vectors in our laboratory) and irradiated feeder autologous splenocytes (1∶1 ratio). After 48–72 hrs, the plates were washed three times with PBS and three times with 0.05% PBS/Tween 20. Then, 2.0 µg/ml biotinylated mouse cytokine-specific mAb (BD Biosciences, San Diego, CA) in PBS/BSA/Tween 20 was added to the wells. After an O/N incubation at 4°C, the plates were washed three times, and HRP-labeled streptavidin (BD Biosciences, San Diego, CA) diluted 1/100 in PBS/BSA/Tween 20 was added to the wells. After 2 hrs, the plates were developed and spots were analyzed by ImmunoSpot Series I analyzer (Cellular Technology, Shaker Heights, OH). The results were expressed as spots per million cells ± SE. Any spots obtained by culturing T cell lines with antigen presenting cells alone without ColV or human Kα1T were subtracted from the number of spots in the test cultures.

### Myeloperoxidase (MPO) Assay

Extracts were prepared by sonication of 100 mg of lungs harvested on days 15 and 30. Neutrophil activity was determined by MPO assay as described earlier [54]. The data was represented as mean ±SEM for 5 different measurements.

### ELISA

ELISA plates (Nunc, Rochester, NY) were coated with ColV or Kα1T (1 µg/ml) in PBS overnight at 4°C [54]. Serum samples were collected from mice treated with anti-MHC class II Ab or isotype Ab. Serum samples were tested (1∶250 and 1∶500) for binding to ColV. Detection was done with anti-mouse IgG, IgM-HRP (Santa Cruz Biotechnology, Santa Cruz, CA), and development using tetramethylbenzidine substrate and read at 450 nm. A sample was considered as positive if the values were over an average cutoff values obtained from normal sera from 10 different mice. Concentration of Abs was calculated based on a standard curve using the binding of known concentration of anti-ColV Abs (Santa Cruz Biotechnology, Santa Cruz, CA).

### Gene expression assay

The PCR was performed to determine gene expression of MHC class II molecule and other interleukins by reverse transcription of mRNA obtained from harvested lungs. Total RNA was extracted from 0.5 mg of lung using TRIzol reagent (Sigma–Aldrich, St Louis, MO). RNA samples were quantified by absorbance at 260 nm. The RNA was reverse-transcribed and PCR was performed in a final reaction volume of 50 µL. Primers used were: I-A^α^ forward, 5′-AAATCCACCCCAGCTACCAAT-3′, I-A^α^ reverse, 5′-GCTGACCCAGCAGCACAG-3′ [55]; 18S rRNA forward, 5′-CTTAAAGGAATTGACGGAAG-3′ and reverse, 5′-TCCGCAGGTTCACCTACGGA-3′; β-actin forward, 5′- ATCGTGCGTGACATCAAAGAGA-3′ and reverse, 5′ -CCACAGGATTCCATACCCAAGA-3′; TGF-β forward, 5′-GATCCCCCTAGCCTCTCATC-3′ and reverse, 5′-GCACAAGCAGGATTGAGTGA-3′; IL-6 forward, 5′-AGTTGCCTTCTTGGGACT-GA-3′ and reverse, 5′-CAGAATTGCCATTGCACAAC-3′; IL-1β forward, 5′-AAACAGATGAAGTGCTCCTTCCAGG- 3′ and reverse, 5′TGGAGAACACCACTTGTTGCTCCA- 3′ and IL-23p19: primers 5′-AGCGGGACATATGAATCTACTAAGAGA-3′, 5′-GTCCTAGTAGGGAGGTGTGAAGTTG-3′. The growth factor expression was analyzed by growth factor RT^2^-PCR profiler™ (SA Biosciences, Frederick, MD). Each sample was analyzed in triplicate. Cycling conditions consisted of an initial denaturation of 95°C for 15 min, followed by 40 cycles of 95°C for 30 s, followed by 61°C for 1 min.

### Flow cytometry

Expression of individual cell specific markers proteins was analyzed by flow cytometry. Splenocytes and lung infiltrating cells were collected by collagen digestion of harvested spleen and lungs, respectively. The specific cellular infiltration was quantitated using appropriate fluorescent tagged Abs for CD4, CD11c, F4/80, CD206, IL-1β, TGF-β, IL-6 and IL-23 (Santa Cruz Biotech, CA), was performed prior to treatment with primary Abs, and then by fluorescent tagged secondary Ab as per manufacturer's protocol (BD Biosciences, Sparks, MD). Cells were analyzed on a LSRII flow cytometer (BD Biosciences) using FACSDiva software. All measurements were taken for 5,000 events that are a display of relative cell count. The data was represented as a mean ± SEM over a 5 different measurements.

### Adoptive transfer

To determine the role of macrophages in induction of OAD following ligation of anti-MHC class II, we administered two concentrations of (1×10^5^ or 1×10^4^) of CD11b+ macrophages. These cell populations were positively selected from lung infiltrating cells [56] at day 60 from mice which were administered with MHC class II Abs or control Abs. The cells were intrabronchially administered along with a single dose of MHC class II Ab since it is our contention that some inflammatory event such as that induced by administration of Abs has to be present for the initiation of immune responses leading to immune response to self-Ags. In previous experiments single dose of Ab either anti-MHC class I or II did not induce OAD lesions. Therefore, if OAD lesions were noted following the above mentioned protocol it can be attributed to adoptively transferred cell population. Serum from these mice was collected on day 30 to quantitate the levels of Abs against ColV and Kα1T, as described earlier. Histopathological analysis of the lungs was also performed to determine the levels of cellular infiltration and epithelial hyperplasia by H&E staining. Fibrosis in the lungs was analyzed by trichrome staining.

### Statistical analysis

Morphometric analysis on tissue sections was performed using NIS-Elements BR software (Melville, NY). The statistical analysis is performed either using GraphPad5.0 (LaJolla, CA) or Origin 6.0 (Northampton, MA) and all the data is represented as a mean ± SEM over a 5 different measurements. The significance was established by performing student-t-test and considered significant when *p*-value was determined to be less than 0.05.
